# Paternal effect on genomic activation, clinical pregnancy and live birth rate after ICSI with cryopreserved epididymal versus testicular spermatozoa

**DOI:** 10.1186/1477-7827-7-142

**Published:** 2009-12-03

**Authors:** Nina Desai, Faten AbdelHafez, Edmund Sabanegh, James Goldfarb

**Affiliations:** 1 Cleveland Clinic Fertility Center, Department of Obstetrics-Gynecology, Cleveland Clinic Foundation, Beachwood, OH, USA; 2 Glickman Urological and Kidney Institute, Cleveland Clinic Foundation, Cleveland, OH, USA

## Abstract

**Background:**

This study takes an in depth look at embryonic development, implantation, pregnancy and live birth rates with frozen epididymal and testicular sperm from obstructed (OA) and non-obstructed (NOA) patients.

**Methods:**

Paternal effect of sperm source on zygote formation, embryonic cleavage, and genomic activation were examined. Additional outcome parameters monitored were clinical pregnancy rate (CPR), implantation rate (IR) and live birth rate.

**Results:**

In this report, we retrospectively analyzed 156 ICSI cycles using cryopreserved epididymal sperm (ES; n = 77) or testicular sperm (TESE; n = 79). The developmental potential of embryos did not appear to be influenced by the type of surgically retrieved sperm. The average number of blastomeres observed on Day 3 was not different among different groups; 7.5 +/- 1.7 (ES), 7.6 +/- 2.1 (TESE-OA) and 6.5 +/- 2.3 (TESE-NOA). Compaction and blastulation rates, both indicators of paternal genomic activation, were similar in embryos derived from ICSI with ES or TESE from OA as well as NOA men. The only parameter significantly affected in NOA-TESE cases was the fertilization rate. CPR and IR with cryopreserved TESE (TESE-OA 59%, 34%, and TESE-NOA 37%, 20%) were also not statistically different, from that achieved with cryopreserved ES (61% and 39%). Live birth rates also appeared to be independent of sperm type. The 87 clinical pregnancies established using cryopreserved TESE and ES, resulted in the birth of 115 healthy infants. No congenital anomalies were noted.

**Conclusion:**

Zygotic activation seems to be independent of sperm origin and type of azoospermia.

## Background

Male factor infertility represents almost one third of infertility cases, of which azoospermia may comprise up to10% of cases [1]. Azoospermia can be either obstructive (OA) or non-obstructive (NOA). In OA there is a blockage in the reproductive pathway of sperm. In such cases, sperm can be retrieved from the epididymis or the testis. With NOA, spermatogenesis itself is impaired and sperm is not usually obtainable from the epididymis necessitating testicular sperm retrieval [2-5]. In severe cases, there may only be scattered foci of sperm production and more extensive biopsy may be necessary to recover tubules with sperm.

Paternal contribution may affect fertilization, early embryo development, genomic activation and ultimately clinical pregnancy. The sperm centrosome is directly involved in forming the sperm aster and organizing the mitotic spindle and plays an important role in early embryonic development (reviewed in [6]). Microtubules, extending from centrioles, are responsible for proper pronuclei movement and fusion [7], as well as pronuclei alignment. Certain types of male infertility and fertilization arrest may be related to the sperm centrosome [8]. Data have suggested an association between centriolar defects and sperm immotility or non progressive motility [9].

Severe male factor cases requiring surgical retrieval of sperm from the epididymis or testis present a unique opportunity to further study the paternal influence on embryonic development. Paternal genome expression and resultant zygotic activation occurs at the 4-8 cell stage in human embryos [10]. A question of obvious interest to reproductive specialists in treating males with severe azoospermia is whether the source of sperm or type of azoospermia affects embryonic development and subsequent outcomes.

This study takes an in depth look at embryonic development and clinical outcomes with frozen epididymal (ES) and testicular (TESE) sperm from OA and NOA patients coming through our IVF program. Paternal genome activation in ICSI embryos is gauged using embryonic compaction and blastulation as morphologic indicators of the onset of zygotic transcription. We also present live birth data from pregnancies established with cryopreserved surgically retrieved sperm.

## Methods

### Study population

We retrospectively analyzed 156 consecutive ICSI cycles performed at the Cleveland Clinic using cryopreserved ES and TESE during the period from January 2001 to December 2007. Azoospermia was classified as OA or NOA based on history, physical examination, FSH levels and surgical findings by the urologist. Specifically, patients with normal testicular volume and ductal anatomy in conjunction with normal gonadotropin levels or a history of known genital tract obstruction such as vasectomy were classified as OA. In addition, patients with a prior normal diagnostic testicular biopsy were assigned to the OA group. Patients with reduced testicular volume and elevated gonadotropin levels or prior testis biopsy revealing compromised spermatogenesis were classified as NOA. All NOA patients received standard genetic testing with karyotype and y-linked microdeletion analysis. All results were normal in our study population. The data for this study was collected via retrospective chart review, in accordance with guidelines of our Institutional Review Board.

### Epididymal and testicular sperm preparation and cryopreservation

Male partners had sperm extracted and cryopreserved prior to IVF cycle start. ES were extracted using either percutaneous sperm aspiration or micro-surgical epididymal sperm aspiration technique. The specimen was assessed under an inverted microscope at 300×. A small 5 μl aliquot was placed on a petri dish and overlaid with oil. Total motile sperm per high powered field (hpf) were counted. At least 10 fields were evaluated. If no sperm were seen in the initial sampling, additional aliquots were examined.

Testicular sperm extraction procedures were performed by the reproductive urologist at our ambulatory surgical unit. Sperm was extracted from testicular tissue by homogenization with a Kontes pestle and release of sperm into modified human tubal fluid medium (m HTF, Irvine) supplemented with10% human serum albumin (HSA, SAGE). Multiple samples from different sites within one or both testes were taken, until either sperm were observed or else the operating surgeon determined that no more tissue could be safely removed. The number of motile sperm per hpf was recorded for each sample.

For cryopreservation, ES and TESE samples were diluted 1:1 with test yolk buffer-glycerol cryoprotectant (Irvine) and aliquotted into cryovials. Vials were vapor frozen for 30 minutes prior to immersion in liquid nitrogen. Based on number of sperm viewed per hpf, 4-8 vials were frozen per patient, each being sufficient for a single IVF cycle. A separate 25 μl "test" aliquot was frozen to assess post-thaw survival. If no motile sperm were observed on test-thaw, the patient was counseled. The option of donor sperm back-up and oocyte freezing were discussed.

### Ovulation induction and oocyte retrieval

Down regulation with leuprolide acetate (Lupron; TAP, Pharmaceuticals, Lake Forest, IL) followed by ovulation induction with gonadotrophins was the primary stimulation protocol utilized. Human chorionic gonadotrophin (hCG) was administered when at least two follicles measured 18 mm in diameter. Oocyte retrieval using trans-vaginal ultrasound guided follicle puncture was performed 36 hours later. The luteal phase was supported with intra-muscular progesterone (50 mg/ml). Cumulus-oocyte complexes were cultured in microdrops of HTF medium (LifeGlobal, Guilford, CT) supplemented with 10 mg/ml of HSA under an oil overlay. Culture dishes were incubated at 37°C with 5.5% CO_2_.

### Thawing of epididymal/testicular sperm and ICSI

Cumulus-oocyte complexes were briefly exposed to hyaluronidase (SAGE) to facilitate removal of the surrounding cumulus cells and to allow evaluation of oocyte nuclear maturity. Mature metaphase II oocytes were injected with thawed epididymal or testicular sperm between 3 and 8 hours after the oocyte retrieval.

Frozen epididymal and testicular sperm specimens were thawed at room temperature and examined for the presence of motile sperm. Specimens with at least one motile sperm per hpf were washed by centrifugation at 200 g for 10 minutes. Sperm pellet was re-suspended in 50-100 μl of mHTF with 10 mg/ml of HSA. Centrifugation was avoided in cases with very low numbers of motile spermatozoa. In these cases the sample was serially diluted to remove the cryoprotectant and facilitate sperm identification and isolation. Motile or twitching sperm were preferentially selected for ICSI. If enough motile or twitching sperm could not be isolated after several hours of searching, sperm with distinctly flexible tails were sometimes considered for injection.

Attention was paid to sperm head morphology during the selection process. Sperm heads were cleared of any surrounding debris by repeated aspiration and expulsion from the ICSI needle to allow clear visualization and prevent inadvertent injection of motile sperm with tiny, irregular or pin heads. The sperm were collected in a media drop in the ICSI dish and moved to 10% polyvinylpyrolidone (PVP, Medicult, Denmark) prior to injection. Time spent by sperm in the PVP drop prior to injection was minimized.

### Embryo evaluation and transfer

Fertilization check was performed 18-20 hours after the ICSI procedure. Normally fertilized zygotes exhibiting two pronuclei were subsequently moved to microdrops containing HTF supplemented with 10% Synthetic Serum Substitute (SSS; Irvine, CA) and cultured for an additional two days. Embryo cleavage and development were monitored daily. Global blastocyst medium with 10% SSS was used for extended culture to the blastocyst stage.

Embryo transfer was generally performed on Day 3 using a Wallace catheter under ultrasound guidance. Embryo selection for Day 3 transfer was based on morphologic parameters. Embryos were graded on the basis of cell number, regularity of blastomeres, good blastomere expansion, fragmentation level and signs of embryonic compaction or increased cell:cell adherence.

In 13 of the cycles studied, total zygote number and embryo quality were high, allowing a shift to a Day 5 transfer. Embryo selection on Day 5 was based on blastocyst maturity, trophectoderm and ICM differentiation [11]. Non-transferred embryos were either frozen on Day 3 or placed in extended culture and frozen on Day 5 or 6 at the blastocyst stage.

Pregnancy testing was performed 15 days after the embryo transfer. Clinical pregnancy was confirmed by the presence of a fetal heart on ultrasound examination at six to eight weeks of pregnancy. Live birth and delivery information was obtained from clinical records.

### Data collection/statistical analysis

The primary outcome measures tabulated were fertilization rate, cleavage rate, Day 3 cell number, compaction, blastocyst formation, number of embryos transferred, clinical pregnancy rate and implantation rate. The implantation rate was calculated by dividing the number of fetal heart tones by the total number of embryos transferred. Multiple pregnancy and live birth rates were also monitored. Results are expressed as means ± SD for numeric variables. Categorical variables were expressed as proportions (%). Mean values were compared using the one way-ANOVA test and the Student's t-test for numeric variables. Proportions were compared using the chi-square test or the Fisher's exact test, depending on sample size. P-values of less than 0.05 were considered to be statistically significant.

## Results

We retrospectively analyzed 156 consecutive IVF cycles performed at the Cleveland Clinic and utilized frozen-thawed ES or TESE for ICSI of mature oocytes. Cryopreserved-thawed ES were used in 75 cycles; while 79 ICSI cycles were performed using cryopreserved TESE. Neither the female (31.9 ± 4 and 32.8 ± 3.6) nor the male (39 ± 8.6 and 38.5 ± 7.0) ages differed significantly between the ES versus the TESE treatment groups, respectively. Male diagnosis for azoospermia and surgical sperm extraction is shown in Table 1. OA accounted for 75% of the cycles in the TESE group. In the ES treatment group, all but one case was OA.

**Table 1:** Azoospermia and male diagnosis

Male Diagnosis	Epididymal	TESE (OA)	TESE (NOA)
**Total Males**	**59**	**44**	**17**
Vasectomy	26	12	
Congenital bilateral absence of vas deferens	16	6	
Ejaculatory dysfunction	3	1	
Infective/inflammatory	2		
Non infective	3	2	
Idiopathic	8	23	3
Germ cell aplasia	1		3
Incomplete maturation arrest			2
Mixed germ cell aplasia and maturation arrest			2
Hypospermatogenesis			5
Testicular carcinoma			1
Atrophy of seminiferous tubules			1

* None of the NOA patient had Klinefelter's syndrome or chromosome Y microdeletions

Table 2 summarizes results from cases using frozen ES versus TESE sperm. The average number of oocytes injected with sperm was 11.2 ± 5.5, 10.8 ± 6.0 and 9.8 ± 4.0 in cycles using ES and TESE sperm from OA and NOA men, respectively (Table 2). Fertilization rate was significantly lower in the TESE-NOA cases (50% vs 66% TESE-OA and 65% ES).

**Table 2:** Comparison of cycle outcome parameters after ICSI with epididymal or testicular sperm from males with obstructive (OA) and non-obstructive (NOA) azoospermia

Sperm Type	Epididymal	Testicular Obstructive OA	Testicular Non Obstructive NOA	P value
Number of retrieval cycles	77	59	20	-
Number of transfers	75	58	19	-
Male partner age	39.0 ± 8.6	39.3 ± 7.5	36.0 ± 4.8	NS
Maternal age	31.9 ± 4.0	32.9 ± 3.5	32.3 ± 4.0	NS
Average number of oocytes	14.0 ± 5.8	14.2 ± 6.2	13.5 ± 7.3	NS
Average oocytes injected	11.2 ± 5.5	10.8 ± 6.0	9.8 ± 4.0	NS
Average oocytes fertilized	7.2 ± 4.4	7.3 ± 4.0	4.9 ± 4.0	0.06
Fertilization rate	65%	66%	50%^a^	< 0.001
Cleavage rate	97%	99%	97%	NS
Average embryos transferred	2.2 ± 0.6	2.5 ± 0.7^b^	2.2 ± 0.8	< 0.05
Average cells on Day 3	7.5 ± 1.7	7.6 ± 2.1	6.5 ± 2.3	NS
Clinical pregnancy rate	61%	59%	37%	NS
Implantation rate	39%	34%	20%	NS
Deliveries (n)	46	33	7	-
Singleton (n)	32	22	6	-
Twins (n)	12	10	1	-
Triplets (n)	2	1	0	-
Boys (n)	31	23	5	-
Girls (n)	31	22	3	-
Miscarriages (n)	0	1	0	-

^a ^Significantly different from ES and TESE-OA

^b ^Significantly different from ES and TESE-NOA

The primary limitation to successful ICSI was the identification and isolation of sufficient numbers of motile and/or twitching sperm from the thawed specimen. Injection of clearly motile sperm, exhibiting slight head movement or twitching was essential for success. In this series, we completely failed to find motile sperm in two cycles, one with ES and the other with TESE sperm from a NOA patient. Injection of immotile sperm did not result in fertilization. Cell number on Day 3, in the cohort of embryos selected for transfer did not differ significantly with sperm type used for ICSI (ES: 7.5 ± 1.7; TESE-OA: 7.6 ± 2.1 and TESE-NOA: 6.5 ± 2.3). Embryos were carefully monitored for signs of increased cell:cell adherence and compaction as a part of the selection process for transfer. We found that approximately 11% of non-transferred embryos within both groups were of a quality suitable for freezing on Day 3. Remaining supernumerary embryos were cultured for 2-3 additional days and monitored for compaction and blastocyst development (Table 3). ICSI with ES or TESE sperm from OA and NOA men resulted in similar rates of embryonic compaction and blastulation. These data suggest that paternal genome activation was not influenced by either the source of sperm or the type of azoospermia.

**Table 3:** Development of embryos not transferred or frozen on Day 3

	Epididymal	Testicular OA	Testicular NOA
**Number of embryos**	**349**	**224**	**48**
**Embryos undergoing compaction**	**74%**	**69%**	**79%**
**Embryos undergoing blastulation**	**38%**	**42%**	**38%**

Comparison of embryos derived from ICSI with epididymal sperm and testicular sperm (obstructive azoospermia;OA) and non-obstructive azoopermia (NOA). Development expressed as percentage of total embryos left in extended culture. No significant differences were observed.

Clinical pregnancy was achieved by 46 couples (61%), 34 couples (59%) and 7 couples (37%) using ES, TESE-OA and TESE-NOA, respectively. Eight patients using thawed ES for ICSI were transferred on Day 5 and seven became pregnant. Day 5 transfers in the TESE group were performed for 5 patients and all 5 achieved a clinical pregnancy. The average number of embryos transferred was significantly higher with TESE-OA cases (2.5 ± 0.7 versus TESE-NOA 2.2 ± 0.8 and ES 2.2 ± 0.6; P < 0.05). The implantation potential of embryos arising from TESE sperm in OA cases (34%) was not statistically different than that from ES group (39%). Although key outcome parameters, namely clinical pregnancy and implantation rates were lower in the TESE-NOA group, the differences were not statistically significant. Nineteen patients had an implantation failure on their first cycle and 15 successfully achieved a pregnancy on a subsequent IVF cycle (TESE group 7/9; ES group 8/10).

The delivery rate between the groups was similar. Forty six pregnancies were established using frozen ES and resulted in a delivery of 62 healthy infants (32 singletons, 12 sets of twins and 2 triplet pregnancies). In the frozen TESE group, 40 of 41 clinical pregnancies delivered, resulting in the birth of 53 healthy infants (28 singletons, 11 twin and 1 triplet pregnancy). One pregnancy resulted in a spontaneous abortion. No congenital anomalies or major malformations were noted.

## Discussion

This comparative study of ICSI outcomes with cryopreserved ES and TESE sperm from males with OA and NOA provides insight in to the developmental capacity of such embryos and paternal effect on genome activation. The high implantation rate per embryo transferred and the resultant live births attest to the quality of embryos being produced with both types of surgically retrieved sperm.

Azoospermia cases present an opportunity to study paternal effect on genomic activation and clinical outcome parameters. The sperm centrosome is directly involved in forming the sperm aster and organizing the mitotic spindle. Microtubules, extending from centrioles, are responsible for proper pronuclei movement and fusion [7], as well as pronuclei alignment. Both centrioles that are necessary for completion of the fertilization step are contributed by the sperm. Zygote expression of mRNA transcripts has been shown to begin at approximately the 4 to 8 cell stage [10,12]. This represents a critical transition point for human embryos- the point at which the paternal genome is believed to be activated [13]. Disruptions in spermatogenesis might be expected to alter paternal genome expression. One of the earliest morphologic indicators of embryonic genome activation in *in vitro *cultured embryos is increased cell:cell adherence at the 8-cell stage, leading to compaction [14]. The arrest of ICSI embryos at the 5-8 cell, close to the time of genome activation [13,15] and lower blastulation rates have been attributed to sperm defects [16-19].

Limited reports detail embryonic development *in vitro *and clinical prognosis in OA versus NOA TESE cases [20-24]. Data from the present work, suggest that sperm origin; epididymal versus testicular does not significantly affect cell number on Day 3, clinical pregnancy outcome and implantation rates. Patients with NOA did however have significantly lower fertilization rates. This may be indicative of limited foci of sperm production, resulting in lower numbers of motile sperm for selection, along with dysfunctional spermatogenesis in these NOA cases.

Interestingly, embryonic compaction was similar in all three groups (Table 3). About 2/3 of embryos derived from surgically retrieved sperm transitioned past the early cleavage stage and exhibited signs of compaction suggesting that paternal genome activation was independent of the type of azoospermia. Rates of blastocyst formation in supernumerary embryos was also not statistically different (ES 38%, TESE-OA 42% and TESE-NOA 38%), supporting this argument. Balaban et al (2001) using fresh ES and TESE sperm also concluded that the type of azoospermia (OA or NOA) did not appear to influence blastulation [25].

Our findings are in agreement with other published reports observing no impact of NOA on clinical pregnancy and implantation rates [22,25-28]. In contrast, Vernaeve et al [29] obtained lower pregnancy (15% vs 24%) as well as implantation (9% vs 13%) rates in TESE cases with an etiology of NOA versus OA, respectively. A meta-analysis published by Nicopoullos et al. (2004), also suggests impaired implantation rates in NOA cases [30]. Clearly, severity of the spermatogenesis defect, technologic aspects of handling such cases as well as other etiological factors may potentially influence the degree of success with TESE specimens from males with NOA.

The technique for effective utilization of frozen surgically retrieved sperm adopted by our laboratory deserves some mention. Assessment and quantitation of pre and post-thaw motility on a test aliquot in all cases involving surgically retrieved sperm was invaluable in cycle management. The IVF laboratory could then anticipate the difficult cases, arrange for more personnel and extra time for screening. Technical aspects of the ICSI procedure itself were also vital to success when dealing with the extremely impaired specimens. Great care was taken to select sperm with distinct motility or head motion and to avoid injection of sperm with dysmorphisms. These data encourage us to advocate this approach in all but the most extreme cases.

## Conclusion

This study examined not only clinical outcomes with surgically retrieved sperm but also paternal contribution to embryonic development. The observations on embryonic compaction suggest that zygotic activation is independent of sperm origin and type of azoospermia. A limitation of the current study is the number of NOA patients. The relationship between specific male diagnosis and paternal genomic activation in NOA needs to be looked at in a larger data series. Such data may be useful in understanding paternal factors correlating to embryonic arrest especially in cases with severe defects in spermatogenesis. Finally, recognition of the importance of compaction and preferential selection of embryos with this attribute for transfer may play a role in achieving high implantation rates with surgically retrieved sperm.

## Competing interests

The authors declare that they have no competing interests.

## Authors' contributions

ND performed the IVF procedures, analyzed the data and proved the manuscript. FA helped in data analysis and prepared the manuscript. ES carried out the urological procedures and revised the manuscript. JG critically reviewed the manuscript. All authors read and approved the final manuscript.
